# Incident Dementia in a Defined Older Chinese Population

**DOI:** 10.1371/journal.pone.0024817

**Published:** 2011-09-23

**Authors:** Ruoling Chen, Zhi Hu, Li Wei, Ying Ma, Zhuming Liu, John R. Copeland

**Affiliations:** 1 Division of Health and Social Care Research, King's College London, London, United Kingdom; 2 School of Health Administrations, Anhui Medical University, Anhuisheng, China; 3 MEMO, Ninewells Hospital and Medical School, University of Dundee, Dundee, United Kingdom; 4 Division of Psychiatry, University of Liverpool, Liverpool, United Kingdom; Brigham & Women's Hospital -, Harvard Medical School, United States of America

## Abstract

**Background:**

Current knowledge about incident dementia is mainly derived from studies undertaken in the West, showing that dementia is related to older age, low socio-economic status, lack of social network, depression and cardiovascular disease risk factors. We know little about incidence and predictors of dementia in China, where the prevalence is increasing and the patterns of risk factors are different.

**Methods:**

Using a standard interview method, we examined 1526 non-demented people aged ≥65 years who had at least minimal educational level in China in a 7.5-year follow up. Incident dementia was diagnosed by GMS-AGECAT algorithms and psychiatrists.

**Results:**

Age-standardised incidence of dementia was 14.7 per 1000 person-years (95%CI 11.3–18.2 per 1000 person-years). The increased risk was significantly associated with age, female gender (adjusted odds ratio 2.48, 95%CI 1.20–5.13), low educational levels, smoking, angina (2.58, 1.01–6.59) and living with fewer family members. Among participants with low educational level, the increased risk was associated with higher income, and with the highest and lowest occupational classes; adjusted odds ratio 2.74 (95%CI 1.12–6.70) for officers/teachers, 3.11 (1.61–6.01) for manual labourers/peasants.

**Conclusions:**

Our findings of high incidence of dementia and increased risk among people having low education levels but high income suggest a more potential epidemic and burden of dementia populations in China. Maintaining social network and activities and reducing cardiovascular factors in late life could be integrated into current multi-faceted preventive strategies for curbing the epidemic of dementia.

## Introduction

Dementia is a major public health challenge, becoming more common as the global population ages. There is widespread recognition of the immense burden that dementia imposes on individuals, communities and health services. It is estimated that dementia contributes 11.2% of years lived with disability in people aged 60 years and older, more than stroke (9.5%), musculoskeletal disorders (8.9%), cardiovascular disease (5.0%), and all forms of cancer (2.4%).[1] Studies in the West have shown that dementia is related to older age, low educational levels, high cardiovascular disease risk factors,[2] and depression. [3], [4] In many western populations, however low socioeconomic status, high cardiovascular risk factors and depression tend to co-occur,[5] making their individual contribution to the cause of dementia difficult to unravel.

With an estimated population of 1.3 billion, China has the largest number of people with dementia in the world.[1] Since its reform in 1978 China has experienced rapid economic growth and increase in life expectancy and the population is ageing. [6] By contrast with western populations, older people in China exhibit different patterns of risk factor clustering with extremes of absolute deprivation [6] combined with high levels of social support, [7] low levels of depression [8], [9] and low levels of cardiovascular disease risk factors (e.g. body mass index) except high blood pressure. Studying such a population will offer insights applicable to the aetiology and prevention of dementia. In this paper, we investigated incidence and predictors of dementia in older people in China.

## Methods

### Participants and Baseline Investigation

Participants were derived from our Anhui cohort study. The methods of its baseline investigation have been fully described before. [7], [9] In brief, we randomly selected 1810 older people aged ≥65 years who had lived in Yiming sub-district of Hefei city, and 1709 aged ≥60 years from all 16 villages in Tangdian district of Yingshang County in 2001 and 2003 respectively. Three thousand, three hundred and thirty six subjects participated in the study (1736 urban participants), with a response rate of 94.8%. Permission for interview and written informed consent were obtained from each elder but if that was not possible, from the closest responsible adult. Refusals were respected. They were interviewed by a trained survey team from the School of Health Administration, Anhui Medical University (*wave 1*). The main interview materials were the Geriatric Mental State (GMS) questionnaire [10] and a general health and risk factors record. [7] According to standard procedures, [11], [12] we measured systolic and diastolic blood pressure, and weight and waist circumference for all participants.

### Follow up of the cohort

Using the same protocol above, we re-examined 2608 cohort members (*wave 2*), one year after the baseline investigation. The response rate was 86.9% after excluding those deceased and who moved to a new home without trace or left home for a long time. During 2007 to 2009, we completed a *wave 3* interview of 1757 surviving cohort members using the GMS and health and risk factors questionnaires. The response rate at *wave 3* was 82.4% based on wave 2 surviving participants. Of 1757 participants, 535 were further interviewed using the Community Screening Instrument for Dementia (CSI-D) and the modified Consortium to Establish a Registry for AD (CERAD) ten-word list learning task with delayed recall. [13] In 8 months (±2 months) after the *wave 3* survey, 4 consultant psychiatrists from Anhui Medical University Teaching Hospital and local mental health hospitals re-interviewed 311 participants (250 with a potential cognitive impairment identified from data of the GMS, [10] the CSI-D and the modified CERAD ten-word list learning task with delayed recall,[13] and 61 controls). We determined vital status of the cohort members and identified causes of death through electronic registration databases from the local Centers for Disease Control and records from the local resident committees. We used a standard Verbal Autopsy questionnaire to explore further causes of death. Six hundred and one deaths were identified.

Ethical approval for the study was obtained from the Research Ethics Committee, University College London.

### Assessment of dementia

A computer program assisted diagnosis–the Automated Geriatric Examination for Computer Assisted Taxonomy (AGECAT) was used to analyse the information from the GMS to identify the principal mental disorders in these participants. [10] AGECAT was developed using a theoretical model and tested against its success at replicating diagnoses on samples diagnosed by psychiatrists. It attempts to replicate the process by which a psychiatrist achieves first, a syndromal diagnosis followed by a differential diagnosis. GMS symptoms are coalesced into a hundred and fifty “symptoms components”. In stage I the symptom components are brought together into groups which typify the major symptom areas of each diagnostic syndrome. The scores on these individual groups determine the final syndromal level of “confidence of diagnosis”. Thus the system uses both quantitative and qualitative measures when allotting subjects to the levels of confidence, and required for its construction many hundreds of clinical decisions on the placement of groups of symptoms components on the syndrome levels. Individual participants are allocated to levels of confidence of diagnosis (0–5) on each of the eight diagnostic syndromes: organic disorder, depression, mania, schizophrenia and paranoid, obsessional, phobic, hypochondriacal, and general anxiety. In stage II the various syndrome levels are compared one with another to derive a final differential diagnosis and a level of confidence of diagnosis from 0–5.

A level of ≥3, in most circumstances designates a “case level” which has been shown to correspond with what psychiatrists usually recognize as a case for intervention. Levels 1 and 2 are designated as “sub-cases”, while level 0 (no confidence level on any syndrome) is classified as “well”. [14] GMS-AGECAT dementia “case” diagnoses have been compared with psychiatrists' diagnoses and DSM III criteria, and applied with good levels of agreement in a variety of settings,[10] including overseas Chinese [15] and elders in China. [16], [17] The GMS-AGECAT diagnosis has been the most widely used international community-based study method for investigations of mental disorders in older people.[10]

For those cohort members who died in the follow up before re-interviewing, we determined dementia caseness from the causes of death. To increase the study power, we included dementia patients from the case-control study diagnosed by psychiatrists after *wave 3* survey.

### Risk factors

The general health and risk factors record [7] contained (1) socio-demographic information, including educational level, main occupation status and annual personal income, smoking and alcohol habits, (2) social support and relationships, (3) psychosocial aspects, (4) doctor-diagnosed cardiovascular diseases and medications and self assessed physical health, (5) adverse life events occurring in the last two years, and (6) hobbies and activities of daily living (ADL).[14]

### Statistical analysis

We restricted our data analysis to participants who had at least minimal educational levels at baseline (n = 1637, out of 3336). This is because (1) the GMS-AGECAT dementia diagnosis was developed for older populations with literacy in the West, and elders in developing countries who were illiterate may have an over-diagnosis of dementia, [16], [17] and (2) we wished to ensure that our data would be comparable to those in the West. [18] To make the urban and rural data more comparable within this study, we excluded rural participants with baseline aged <65 years (n = 70). Thus, 1526 elders who were free of dementia at baseline after excluding 41 patients with baseline dementia were followed up for this study. We computed person-years at risk (PYARs) of the cohort members to the end of follow up, date of dementia ascertainment, death or losing follow up. The incidence rate of dementia cases with 95% confidence intervals (CIs) among men and women was calculated and were age standardised by the world population of 2002–2009 (www.census.gov). Since the incidence rate of dementia is low, [19] where odds ratio (OR) can be used to estimate a relative risk, we employed a logistic regression model, with adjustment for age and sex, to investigate the associations of baseline risk factors with incident dementia. A multivariate logistic regression model, which included all variables with p≤0.100 in the age-sex adjusted analysis, was used to further explore the independent effects of risk factors. Due to the rapid economic reform of the 1980s in China, socioeconomic factors of educational level, occupational class annual incomes may not be highly related; for example a few Chinese (eg, those running business) have become rich rapidly, without basic education level. Thus we further examined the combined effects of these socioeconomic factors on dementia. All analyses were performed using the SPSS statistical package (Windows version 16.0; SPSS Inc., Chicago, Illinois).

## Results

Among 1526 non-demented participants, 1307 were followed up (of which 1238 were re-interviewed at *wave 2* or *wave 3*). Up to 7.5 years follow up (median 3.9 years), there was a total of 5083.1 PYARs and 80 cases of dementia occurred (63 dementia diagnoses from GMS-AGECAT, 15 from psychiatrists and 2 from the underlying causes of death). Table 1 shows their number and incident rate by age and sex. World age-standardized incidence of dementia per 1000 person years at risk was 14.7 (95%CI 11.3–18.2) (in men 10.9, 6.7–15.0; in women 19.8, 13.5–26.0). The incident rate increased with lower educational levels; 7.2 (4.0–10.4) per 1000 PYARs, 13.3 (7.4–19.3) and 32.6 (20.6–44.52) among participants having educational levels of ≥ high school, secondary school, and primary school respectively. The incidence of dementia was 13.4 (10.0–16.8) in urban elders and 25.0 (10.7–39.3) in rural.

**Table 1:** Incidence of dementia diagnosed by the GMS-AGECAT in older people in Anhui, China: participants, number of cases and rate by age and sex.

Age groups,	Nos. of Person	Dementia cases	
y	Years at risk	Nos.	Rate per 1000 (95%CI)
Men			
65–69	408.0	3	7.4 (1.5–21.3)
70–74	1022.8	5	4.9 (1.7–11.4)
75–79	849.6	12	14.1 (7.3–24.5)
≥80	594.9	16	26.9 (15.4–43.3)
* All ages*	*2875.3*	*36*	*12.5 (8.7*–*17.3)*
Women			
65–69	461.7	4	8.7 (2.4–22.0)
70–74	883.1	16	18.1 (10.4–29.2)
75–79	536.5	12	22.4 (11.7–38.8)
≥80	326.4	12	36.8 (19.1–63.4)
* All ages*	*2207.8*	*44*	*19.9 (14.5*–*26.7)*
Total			
65–69	869.7	7	8.0 (3.2–16.5)
70–74	1906.0	21	11.0 (6.8–16.8)
75–79	1386.1	24	17.3 (11.2–25.7)
≥80	921.3	28	30.4 (20.3–43.6)
* All ages*	*5083.1*	*80*	*15.7 (12.3*–*19.2)*

Table S1 gives the frequencies of risk factors and the age- and sex- adjusted ORs for incident dementia. In these older people, 66.4% of participants had a mean of annual personal income of US$ 1055, with further 15.7% having income at US$ 689 and lower. Risk of dementia increased with older age and female gender. Age-sex adjusted odds ratio significantly increased with rurality, lower educational level, lower occupation status, serious financial problems, smoking and psychosocial factors. It had borderline significances for a lower BMI, the highest income, angina, no hobbies of painting/playing chess/flower planting/pet, and living with fewer family members. The risk of dementia was not significantly related to other factors listed in Table S1. In a multivariate logistic regression model, the increased risk of developing dementia was significantly and independently associated with older age, female gender, lower educational level, smoking, angina and living with fewer family members (table 2). The relationship between hypochondriasis and dementia was borderline significant.

**Table 2:** Multivariate analysis for incident dementia in older people in Anhui, China.

Variable†	Multiple adjusted analysis		
	OR	95%CI	P
***Basic characteristics***			
**Age (years)**	1.04	(1.00–1.09)	0.047
**Sex**			
Men	1.00		
Women	2.48	(1.20–5.13)	0.014
Body mass index (**kg/m^2^)**			
<20	1.00		
20–<23	0.96	(0.47–2.00)	0.922
23–<26	0.63	(0.30–1.33)	0.223
≥26	0.45	(0.20–1.05)	0.066
**Urban-rurality**			
Urban	1.00		
Rural	0.91	(0.14–5.70)	0.916
***Socio-economic position, lifestyles, and hobby***			
***Educational level***			
≥High secondary school	1.00		
Secondary school	1.35	(0.70–2.61)	0.365
Primary school	2.12	(1.03–4.38)	0.042
***Main occupation***			
Officer/teacher	1.00		
Businessmen/non-labouring worker	0.60	(0.18–1.94)	0.389
Manual labourer	1.93	(0.97–3.87)	0.063
Peasant	1.33	(0.60–2.99)	0.483
***Annual income***			
Very satisfactory	1.13	(0.56–2.24)	0.737
Satisfactory	1.00		
Average	0.53	(0.21–1.37)	0.190
Poor or Financial problems	0.87	(0.27–2.80)	0.809
**Smoking habits**			
Never-smoking	1.00		
Ex-smoking	1.66	(0.53–5.16)	0.383
Current-smoking	2.39	(1.21–4.72)	0.012
**Painting/playing chess/flower planting/pet**			
Yes	1.00		
No	1.16	(0.67–1.98)	0.599
**Angina**			
No	1.00		
Yes	2.58	(1.01–6.59)	0.047
***Social network***			
**Good relation with others, ease in acquiring friends**			
Yes	1.00		
No	0.90	(0.38–2.13)	0.814
***Living with***			
No-one/Others	1.00		
Spouse only or Parents only	0.69	(0.35–1.36)	0.282
Children and/or Grant children only	0.55	(0.23–1.33)	0.185
Spouse and/or grand/children and/or parents	0.36		0.012
***Psychosocial factors***		0.16–0.80)	
**Worrying**			
No	1.00		
Yes	1.45	(0.78–2.69)	0.241
**Hypochondriasis**			
No	1.00		
Yes	1.81	(0.90–3.65)	0.095
**Anything (else) severely upsetting**			
No	1.00		
Yes	1.62	(0.55–4.78)	0.385
**Horrifying experience (including, accident, fire, physical attack, etc)**			
No	1.00		
Yes	1.70	(0.62–4.63)	0.302

† including all variables which had significant level < = 0.100 from age-sex adjustment analysis in Table S1, which were listed in table 2.

**Figure 1:**
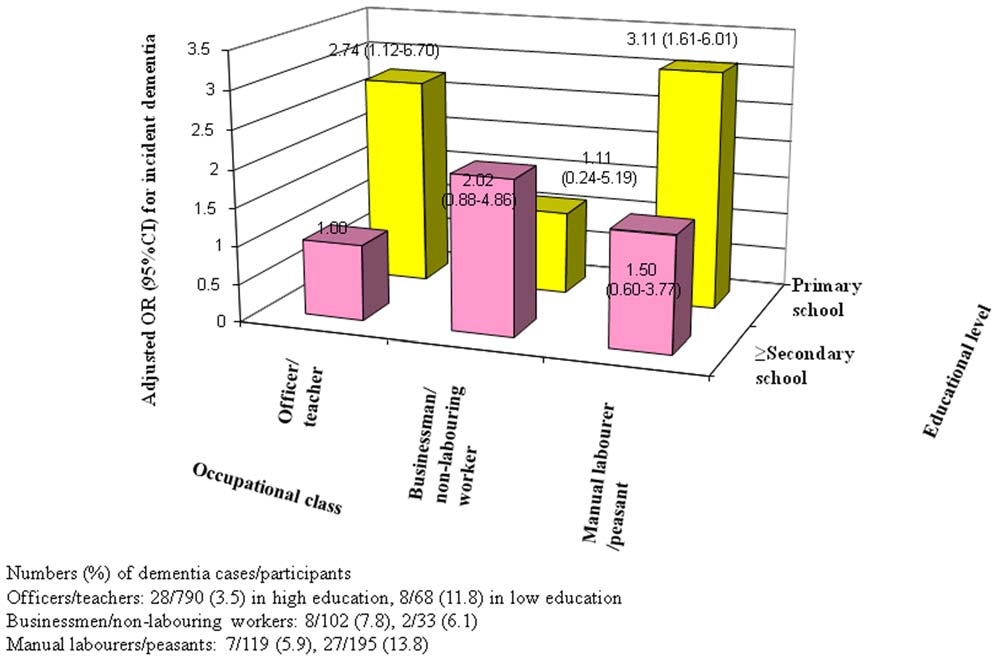
Multiple adjusted odds ratio for incident dementia in the combined occupational class and educational level.

The combined analysis of income and education showed that participants with lower educational level and having higher incomes had a significantly increased risk of dementia (table 3). It is also observed that the risk of dementia significantly increased among participants with lower educational levels but the highest occupational classes (officers/teachers) and the lowest (manual labourers/peasants) (figure 1). In the combinations of income and occupational class, only borderline significant associations were observed; compared to officers/teachers with satisfactory (ie, middle) income, manual labourers/peasants with middle income had a multivariate adjusted OR of 2.04 (0.93–4.47).

**Table 3:** Number of incident dementia and odds ratio (OR) for combined family income and educational level in older people in Anhui, China.

		Educational Level				
	≥Secondary school			Primary school		
Annual income	Nos of case/participants (%)	OR (95%CI) †	p	Nos of case/participants (%)	OR (95%CI) †	p
Very satisfactory	9/172 (5.2)	1.18 (0.52–2.71)	0.693	8/46 (17.4)	2.99 (1.12–7.99)	0.029
Satisfactory	26/682 (3.8)	1.00		13/119 (10.9)	2.66 (1.27–5.56)	0.010
Average, Poor or Financial problems	8/157 (5.1)	0.79 (0.31–2.01)	0.613	16/131 (12.2)	1.40 (0.52–3.74)	0.505

† adjusted for age (continuous), sex, Body mass index (group), urban-rurality, smoking, hobbies, relationship, living with, worrying, hypochondriasis, anything severely upsetting, horrifying experience, and angina.

## Discussion

In an older population in China, we found an increased incidence of dementia, in comparison with some Caucasian populations in the West. [19] The increased risk for developing dementia was associated with older age, female gender, lower educational level, smoking, angina and living with fewer family members. Among participants with low level of education, excess of dementia was associated with higher income.

### Strengths and weaknesses of the study

The main contribution, beyond the intrinsic importance of studying dementia in the world's most populous nation, lies in what it tells us about the incidence rate and predictors of dementia in older people who had different patterns of risk factors compared to those in the West. The population in China which we studied had high levels of absolute poverty, but high levels of social support and low levels of depression and cardiovascular disease, and had experienced rapid economic change in recent years. A second strength is the high response rates of participants at both baseline and follow up to standardized face-to-face interviews. Our study has limitations. First, the cohort data were not large, which gave a wide confidence interval on the risk factors. However, we have identified many important risk factors being statistically significant, including psychosocial factors even after adjustment for age and sex, and our findings are more conservative when the multivariate modelling was applied. Second, we did not carry out a direct validation study of GMS-AGECAT dementia diagnosis in our cohort as it has been validated in other elderly Chinese populations. [15], [17] Our recent data analysis of 5-year all-cause mortality in the cohort (*proceedings in the 175^th^ annual conference of the Royal Statistical Society in Edinburgh, 2009*) showed that the risk of all-cause mortality was associated with the baseline GMS-AGECAT dementia (age-sex adjusted hazard ratio was 2.3, 95%CI 1.3–3.9), which was consistent with those in the West.[20] Thus, the diagnosis of GMS-AGECAT dementia in our study may be validated indirectly. Third, in the GMS-AGECAT dementia diagnosis we did not know the exact date when the incident dementias occurred but recorded them at the survey interview. We therefore analysed data according to the survey date where the casenesses were first identified to calculate person-years. This may have underestimated the incidence rate of dementia slightly, attenuating our findings of the higher incidence of dementia in this population compared to those in some studies in the West. [19] The impact would be similar between men and women, and thus the findings of gender differences in incidence would not be substantially changed. This does not prevent us from making a claim that the incidence of dementia in this population is higher than those in some other studies in the West, and would not affect the findings in the logistic regression analysis. Finally, the study did not include genetics (e.g., APOE 4) for investigation, which would confound the associations of risk factors with dementia. But we included angina in multivariate analysis, which would reduce the confounding effect.

### Incident rate of dementia and gender differences

Prospective follow up studies examining incidence and determinants of dementia in China are scarce. A meta-analysis study in China [21] showed about 50% lower prevalence of dementia than in the West. The lower prevalence estimated may be due to the dementia diagnosis by different instruments and methods, some of which are criticised for the primacy accorded to memory impairment.[22] Using the standard method of diagnosing dementia in the community, we identified that the incidence in this low income older population was higher than that in some in the West, eg, the Rotterdam study suggested the incidence rate of dementia per 1000 person-years is 1.7, 5.1, 15.3 and 33.9 at ages of 65–69, 70–74, 75–79 and ≥80 years, [19] which are lower than these in our study (table 1) except for the age group of ≥80 where our sample included younger participants than that the Rotterdam study and thus the crude rate was not higher. The incidence of dementia in this study is also higher than that among older people who were literate in Beijing [23] and Shanghai [24], which had relatively higher levels of education and occupational class. The increased incidence in this population was mainly from excess dementia in elders with lower level of education, and in women.

The rural participants had a higher risk of dementia than their urban counterparts because of their lower levels of educational level, occupation class and income. They had relatively low socioeconomic levels, particularly in earlier life [7] but had rapid economic improvement from mid- or later life. We previously found such a rural-urban difference in the risk of depression among the participants. [7], [9] The high risk of psychiatric diseases in older population in rural China has been at least partly explained by their low socio-economic status, suggesting an urgent task of reducing health inequality in mental health in older people. Our finding of significant gender differences in the incident dementia was different from some studies in the West (eg, the Rotterdam study showed that overall, dementia incidence was similar for men and women (rate ratio 1.00, 0.80–1.24 women *vs*. men), and only after 90 years of age dementia incidence declined in men but not in women leading a significantly increased rate ratio. [19] Older Chinese women have lower socio-economic status compared to men, particularly at younger ages (e.g., the priority of going to school was given to boys). When analysis was confined to those with ≥ secondary school education, the gender difference was not substantially reduced. The reasons for Chinese women having a higher incidence of dementia need to be further explored.

### Socioeconomic status

The participants in our study suffered a long-term absolute poverty from birth to the end of 1970s, having experienced Guomindan rule (nationalist party), the Japanese invasion, civil war, the Great Leap Forward, 3-year starvation (1960–62) and the Cultural Revolution.[25] Since the economic reform of the 1980s annual incomes have increased, and dietary patterns changed with increased meat consumption. Those richer people may change their lifestyle dramatically, towards westernization. Our baseline data (on request) show that the elders with higher incomes had higher levels of cardiovascular disease (except for stroke) and risk factors (CVDRFs), a pattern opposite to that observed in the West. It is unclear whether these factors could explain an increased risk of dementia in the participants with the highest income. Our recent 4-province study of dementia in China also showed that the highest income was associated with increased risk of dementia among people with lower levels of education and occupation class. [26] In this study, after further adjustment for CVDs we found that the findings were not substantially changed. The reasons for this relationship need to be further explored.

### Cardiovascular disease risk factors and psychosocial factors

In western populations, cardiovascular disease and risk factors are common, and may increase the risk of dementia.[2] The Cardiovascular Health Study Cohort in America showed a borderline significant relationship between angina and dementia (adjusted relative risk 1.3, 1.0–1.7).[27] Yet, in this study levels of overall cardiovascular disease and risk factors among the older population in China were lower than those in western countries. We still found that the increased risk of dementia was related to angina. Prevention, management and treatment of angina may reduce the risk of dementia. Surprisingly, we observed that larger body mass index (BMI) was associated with a lower risk of dementia (trend p = 0.013). A recent study in America [28] has shown that lower baseline BMI was associated with more rapid cognitive decline within one year among people with mild cognitive impairment. The causal relation between lower BMI and increased risk of dementia requires further investigations in large cohort studies with longer-term follow up.

In western countries where depressive disorders are the most common psychiatric condition, many studies [29] but not all [30] have shown that a history of depression or depressive symptoms was a risk factor for dementia. In the current study, although we could not examine incident dementia in relation to baseline depression due to its small number, [7], [9] we did find a significant relationship between depressive symptoms and the risk of dementia in age-sex adjusted analysis but the statistical significance was reduced after adjustment for other factors. To determine the aetiological role of depression and depressive symptoms in dementia, we require larger, long-term follow up cohort studies.

Our studies showed a “dose-response” relationship between living in families and the risk of dementia. The lower incidence of dementia among persons living with more family members is interesting, and is unlikely to result from chance or bias as we interviewed elders personally and any dementia should have been revealed. Living within a big family is a Chinese culture and tradition. Living with more family members may stimulate the brain, improving cognitive function by close contact.

In summary, there is an increasing incidence of dementia in older people in China. Apart from older age, female gender, low educational level and some cardiovascular factors, the increased risk of dementia was related to higher income among elders with low level of education, and living with fewer family members. This has important implications. It has indicated that socioeconomic and psychosocial aspects of Chinese populations may play a role in the epidemic of dementia and need to be considered for future dementia preventive strategy. Living with more family members-a Chinese tradition has been declining in recent decades in China and in other countries. Increasing social network and contacts in order to reverse this trend could help prevent dementia. Further exploration of Chinese culture and tradition and rapid economic growth associated with lifestyle changes may yield insight into preventive factors for dementia, which would be of benefit in both China and Western countries.

## Supporting Information

Table S1 **Distribution of baseline risk factors and odds ratio (OR) for incident dementia in older people in Anhui, China.**
(DOC)
