# Supplementary material for: Incident Dementia in a Defined Older Chinese Population
Source: PLoS One. 2011 Sep 23;6(9):e24817. doi: 10.1371/journal.pone.0024817 (PMC3179466; doi:10.1371/journal.pone.0024817)
Supplement: Table S1 — Distribution of baseline risk factors and odds ratio (OR) for incident dementia in older people in Anhui, China. (DOC) [file pone.0024817.s001.doc]

**Supplement Table. Distribution of baseline risk factors and odds ratio** (OR) for incident dementia in older people in Anhui, China

| Variable | Dementia case N=80 | |  | Non-dementia case N=1227 | |  | Age-sex adjusted analysis† | | |
| --- | --- | --- | --- | --- | --- | --- | --- | --- | --- |
|
|  | n | (%) |  | n | (%) |  | OR | 95%CI | P |
| ***Basic characteristics*** |  |  |  |  |  |  |  |  |  |
| **Age (years)** |  |  |  |  |  |  |  |  |  |
| 65-69 | 26 | *32.5* |  | 512 | *41.7* |  | 1.00 |  |  |
| 70-74 | 22 | *27.5* |  | 395 | *32.2* |  | 1.20 | (0.67-2.15) | 0.550 |
| 75-79 | 16 | *20.0* |  | 210 | *17.1* |  | 1.65 | (0.86-3.16) | 0.130 |
| ≥80 | 16 | *20.0* |  | 110 | *9.0* |  | 3.20 | (1.65-6.23) | <0.001 |
| **Sex** |  |  |  |  |  |  |  |  |  |
| Men | 36 | *45.0* |  | 703 | *57.3* |  | 1.00 |  |  |
| Women | 44 | *55.0* |  | 524 | *42.7* |  | 1.77 | (1.12-2.8) | 0.015 |
| Body mass index **(kg/m^2^)** ^@1^ |  |  |  |  |  |  |  |  |  |
| <20 | 14 | *17.5* |  | 140 | *11.4* |  | 1.00 |  |  |
| 20-<23 | 29 | *36.3* |  | 361 | *29.4* |  | 0.83 | (0.42-1.62) | 0.582 |
| 23-<26 | 24 | *30.0* |  | 423 | *34.5* |  | 0.60 | (0.30-1.20) | 0.148 |
| ≥ 26 | 13 | *16.3* |  | 303 | *24.7* |  | 0.47 | (0.21-1.03) | 0.060 |
| **Urban-rurality** |  |  |  |  |  |  |  |  |  |
| Urban | 66 | *82.5* |  | 1126 | *91.8* |  | 1.00 |  |  |
| Rural | 14 | *17.5* |  | 101 | *8.2* |  | 2.73 | (1.44-5.18) | 0.002 |
| Socio-economic position, lifestyles, and hobby |  |  |  |  |  |  |  |  |  |
| *Educational level* |  |  |  |  |  |  |  |  |  |
| ≥High secondary school | 22 | *27.5* |  | 621 | *50.6* |  | 1.00 |  |  |
| Secondary school | 21 | *26.3* |  | 347 | *28.3* |  | 1.59 | (0.86-2.94) | 0.140 |
| Primary school | 37 | *46.3* |  | 259 | *21.1* |  | 3.44 | (1.97-6.03) | <0.001 |
| *Main occupation* |  |  |  |  |  |  |  |  |  |
| Officer/teacher | 36 | *45.0* |  | 822 | *67.0* |  | 1.00 |  |  |
| Businessmen/non-labouring worker | 10 | *12.5* |  | 125 | *10.2* |  | 1.60 | (0.76-3.36) | 0.213 |
| Manual labourer | 24 | *30.0* |  | 193 | *15.7* |  | 2.54 | (1.46-4.42) | 0.001 |
| Peasant | 10 | *12.5* |  | 87 | *7.1* |  | 2.47 | (1.17-5.21) | 0.018 |
| *Annual income (estimated average)* |  |  |  |  |  |  |  |  |  |
| Very satisfactory (US$1589 ) | 21 | *26.3* |  | 213 | *17.4* |  | 1.69 | (0.98-2.89) | 0.059 |
| Satisfactory (US$1055) | 47 | *58.8* |  | 821 | *66.9* |  | 1.00 |  |  |
| Average (US$ 689) | 9 | *11.3* |  | 163 | *13.3* |  | 0.93 | (0.45-1.95) | 0.851 |
| Poor (US$ 291) | 3 | *3.8* |  | 30 | *2.4* |  | 1.67 | (0.48-5.75) | 0.418 |
| **Serious financial problems in the past 2 years** |  |  |  |  |  |  |  |  |  |
| No | 64 | *80.0* |  | 1101 | *89.7* |  | 1.00 |  |  |
| Yes^@2^ | 16 | *20.0* |  | 126 | *10.3* |  | 2.41 | (1.33-4.38) | 0.004 |
| **Alcohol drinking** |  |  |  |  |  |  |  |  |  |
| Yes | 11 | *13.8* |  | 241 | *19.6* |  | 1.00 |  |  |
| No | 69 | *86.3* |  | 986 | *80.4* |  | 1.27 | (0.64-2.5) | 0.491 |
| **Smoking habits** |  |  |  |  |  |  |  |  |  |
| Never-smoking | 36 | *51.4* |  | 571 | *60.6* |  | 1.00 |  |  |
| Ex-smoking | 5 | *7.1* |  | 87 | *9.2* |  | 1.81 | (0.62-5.3) | 0.277 |
| Current-smoking | 29 | *41.4* |  | 285 | *30.2* |  | 2.84 | (1.51-5.34) | 0.001 |
| **Watching TV** |  |  |  |  |  |  |  |  |  |
| Yes | 73 | *91.3* |  | 1121 | *91.4* |  | 1.00 |  |  |
| No | 7 | *8.8* |  | 106 | *8.6* |  | 0.94 | (0.42-2.11) | 0.884 |
| **Painting/playing chess/flower planting/pet**^@3^ |  |  |  |  |  |  |  |  |  |
| Yes | 37 | *46.3* |  | 734 | *59.8* |  | 1.00 |  |  |
| No | 43 | *53.8* |  | 493 | *40.2* |  | 1.57 | (0.99-2.49) | 0.055 |
| ***Cardiovascular and other health risk factors*** |  |  |  |  |  |  |  |  |  |
| **Hypertension (BP ≥160/95 mmHg or taking antihypertensive drugs)** |  |  |  |  |  |  |  |  |  |
| *“Non”-hypertension* |  |  |  |  |  |  |  |  |  |
| BP<140/90 mmHg | 29 | *36.3* |  | 501 | *40.8* |  | 1.00 |  |  |
| BP ≥140/90 and <160/95 mmHg | 19 | *23.8* |  | 237 | *19.3* |  | 1.38 | (0.75-2.53) | 0.300 |
| *Hypertension* |  |  |  |  |  |  |  |  |  |
| Undetected | 6 | *7.5* |  | 82 | *6.7* |  | 1.08 | (0.42-2.74) | 0.878 |
| Untreated | 2 | *2.5* |  | 30 | *2.4* |  | 1.14 | (0.26-5.05) | 0.866 |
| Uncontrolled | 9 | *11.3* |  | 113 | *9.2* |  | 1.31 | (0.60-2.86) | 0.499 |
| Controlled | 15 | *18.8* |  | 264 | *21.5* |  | 0.99 | (0.52-1.88) | 0.970 |
| **Hypercholesterolemia** |  |  |  |  |  |  |  |  |  |
| No | 72 | *90.0* |  | 1046 | *86.4* |  | 1.00 |  |  |
| Yes | 8 | *10.0* |  | 165 | *13.6* |  | 0.72 | (0.34-1.53) | 0.391 |
| **Angina** |  |  |  |  |  |  |  |  |  |
| No | 72 | *91.1* |  | 1173 | *95.9* |  | 1.00 |  |  |
| Yes | 7 | *8.9* |  | 50 | *4.1* |  | 2.14 | (0.93-4.93) | 0.074 |
| **Heart disease (coronary or other types of heart disease)** |  |  |  |  |  |  |  |  |  |
| No | 59 | *73.8* |  | 955 | *78.2* |  | 1.00 |  |  |
| Yes | 21 | *26.2* |  | 266 | *21.8* |  | 1.21 | (0.72-2.04) | 0.468 |
| **Stroke** |  |  |  |  |  |  |  |  |  |
| No | 77 | *96.3* |  | 1184 | *96.6* |  | 1.00 |  |  |
| Yes | 3 | *3.8* |  | 42 | *3.4* |  | 1.04 | (0.31-3.44) | 0.956 |
| **Diabetes** |  |  |  |  |  |  |  |  |  |
| No | 73 | *91.3* |  | 1104 | *90.1* |  | 1.00 |  |  |
| Yes | 7 | *8.8* |  | 121 | *9.9* |  | 0.89 | (0.4-1.98) | 0.775 |
| ***Other health status*** |  |  |  |  |  |  |  |  |  |
| **Self assessed physical health status** ^@4^ |  |  |  |  |  |  |  |  |  |
| Good | 41 | *51.3* |  | 722 | *58.8* |  | 1.00 |  |  |
| Average | 30 | *37.5* |  | 372 | *30.3* |  | 1.45 | (0.89-2.37) | 0.141 |
| Poor | 9 | *11.3* |  | 133 | *10.8* |  | 1.15 | (0.55-2.44) | 0.711 |
| **Hearing problems** ^@5^ |  |  |  |  |  |  |  |  |  |
| No | 68 | *85.0* |  | 1043 | *85.0* |  | 1.00 |  |  |
| Yes | 12 | *15.0* |  | 182 | *14.8* |  | 1.19 | (0.61-2.31) | 0.605 |
| **Activity of daily living (score)** ^§^ |  |  |  |  |  |  |  |  |  |
| 0 | 68 | *85.0* |  | 1116 | *91.0* |  | 1.00 |  |  |
| 1-4 | 6 | *7.5* |  | 67 | *5.5* |  | 1.18 | (0.48-2.9) | 0.719 |
| ≥5 | 6 | *7.5* |  | 44 | *3.6* |  | 1.76 | (0.70-4.43) | 0.228 |
| ***Social network*** |  |  |  |  |  |  |  |  |  |
| *Marital status* |  |  |  |  |  |  |  |  |  |
| Married | 57 | *71.3* |  | 1010 | *82.3* |  | 1.00 |  |  |
| Widowed/divorced/ Never married | 23 | *28.8* |  | 217 | *17.7* |  | 1.36 | (0.78-2.37) | 0.274 |
| *Living with* |  |  |  |  |  |  |  |  |  |
| No-one | 9 | *11.3* |  | 81 | *6.6* |  | 1.00 |  |  |
| Spouse only | 38 | *47.5* |  | 579 | *47.2* |  | 0.71 | (0.33-1.55) | 0.393 |
| Children and/or Grant children only^@6^ | 10 | *12.5* |  | 128 | *10.4* |  | 0.60 | (0.23-1.56) | 0.294 |
| Spouse and/or grand/children and/or parents | 14 | *17.5* |  | 385 | *31.4* |  | 0.42 | (0.17-1.03) | 0.059 |
| Others | 9 | *11.3* |  | 52 | *4.2* |  | 1.55 | (0.57-4.21) | 0.390 |
| **Frequency of visiting children or other relatives** ^@7^ |  |  |  |  |  |  |  |  |  |
| Daily | 36 | *45.0* |  | 520 | *42.4* |  | 1.22 | (0.73-2.05) | 0.455 |
| At least weekly - < monthly | 27 | *33.8* |  | 489 | *39.9* |  | 1.00 |  |  |
| Monthly | 13 | *16.3* |  | 178 | *14.5* |  | 1.38 | (0.69-2.74) | 0.360 |
| Never | 4 | *5.0* |  | 40 | *3.3* |  | 1.58 | (0.52-4.81) | 0.421 |
| **Good relation with others, ease in acquiring friends** ^@8^ |  |  |  |  |  |  |  |  |  |
| Yes | 71 | *88.8* |  | 1151 | *93.8* |  | 1.00 |  |  |
| No | 9 | *11.3* |  | 76 | *6.2* |  | 1.92 | (0.92-4.01) | 0.083 |
| ***Psychosocial factors*** |  |  |  |  |  |  |  |  |  |
| **Feeling lonely**^@9^ |  |  |  |  |  |  |  |  |  |
| No | 73 | *91.3* |  | 1167 | *95.1* |  | 1.00 |  |  |
| Yes | 7 | *8.8* |  | 60 | *4.9* |  | 1.69 | (0.74-3.89) | 0.216 |
| **Worrying** |  |  |  |  |  |  |  |  |  |
| No | 55 | *68.8* |  | 1003 | *81.7* |  | 1.00 |  |  |
| Yes | 25 | *31.3* |  | 224 | *18.3* |  | 2.04 | (1.24-3.36) | 0.005 |
| **Hypochondriasis** |  |  |  |  |  |  |  |  |  |
| No | 62 | *77.5* |  | 1104 | *90.0* |  | 1.00 |  |  |
| Yes | 18 | *22.5* |  | 123 | *10.0* |  | 2.61 | (1.48-4.58) | 0.001 |
| **Phobias** |  |  |  |  |  |  |  |  |  |
| No | 78 | *97.5* |  | 1212 | *98.8* |  | 1.00 |  |  |
| Yes | 2 | *2.5* |  | 15 | *1.2* |  | 2.00 | (0.44-9.01) | 0.367 |
| **Death of closely related people**^@10^ |  |  |  |  |  |  |  |  |  |
| No | 65 | *81.3* |  | 1063 | *86.6* |  | 1.00 |  |  |
| Yes | 15 | *18.8* |  | 164 | *13.4* |  | 1.57 | (0.87-2.85) | 0.136 |
| **Anything (else) severely upsetting** |  |  |  |  |  |  |  |  |  |
| No | 63 | *78.8* |  | 1104 | *90.0* |  | 1.00 |  |  |
| Yes | 17 | *21.3* |  | 123 | *10.0* |  | 2.68 | (1.49-4.8) | 0.001 |
| **Horrifying experience (including, accident, fire, physical attack, etc)** |  |  |  |  |  |  |  |  |  |
| No | 59 | *73.8* |  | 1077 | *87.8* |  | 1.00 |  |  |
| Yes | 21 | *26.3* |  | 150 | *12.2* |  | 2.72 | (1.58-4.68) | <0.001 |
|  |  |  |  |  |  |  |  |  |  |

† adjustment for continuous variable of age.

^@1^ data shown similar for “Height”, “Weight” and “Waist circumference”

^@2^ data from the combination of financial problems with income shown that the OR were 1.60 (0.88-2.91) for “very satisfactory income”, 0.90 (0.37-2.18) for “average” and 2.64 (1.45-4.82) for “poor income or serious financial problems”.

^@3^ data shown similar for “Walking or group tourist”, “Playing poker/magian/other games” and “Participating in senior community activity for a chat in leisure time”

^@4^ data shown similar for “Health obviously deteriorating in the last two years”.

^@5^ data shown similar for “Vision problems” and “COPD”.

^@6^ data of “Parents only” included 0 participants in Case group and 2 participants in control group, with no significant OR.

^@7^ data shown similar for “Frequency of contacting and speaking to friends in village” and “Frequency of contacting and speaking to neighbours”.

^@8^ data shown similar for “good relationship with neighbours”, “Help available when needed”, and “Worrying about children”.

^@9^ data shown similar for “Optimism”.

^@10^ data shown similar for “Lost valuable things”, “Unpleasant things with relatives, friends or neighbours” and “Anything (else) severely upsetting” .

^§^ the participant reported their level of difficulty in questions of ADL scale. The valid response was ‘no difficulty alone’ (score 0), ‘manages alone with difficulty (score 1) cannot do alone (score 2). The scale consists of 14 items: having a bath or all-over wash, washing hands and face, putting on shoes and stockings/socks, doing up buttons and zips, dressing yourself other than the above, getting to and using the WC, getting in and out of bed, feeding self, shaving (men) or doing hair (women), cutting your own toenails, getting up and down steps, getting around the house, going out of doors alone and taking medicine.
